# Simultaneous laparoscopic cholecystectomy and transabdominal preperitoneal hernioplasty: two case reports evaluate the safety and surgical complications

**DOI:** 10.1002/ccr3.1141

**Published:** 2017-11-09

**Authors:** Shawqi Arafat, Mhd Belal Alsabek

**Affiliations:** ^1^ Department of Surgery Damascus Hospital Damascus Syria; ^2^ Department of Surgery Syrian Private University (SPU) Damascus Syria

**Keywords:** Hernia, laparoscopic cholecystectomy, surgical site infection, transabdominal preperitoneal

## Abstract

The safety of combining transabdominal preperitoneal (TAPP) hernioplasty and cholecystectomy in one laparoscopic surgery is still for many years questionable. We report two cases describing the safety and discussing the main concerns regarding the complications. As there are only few cases discuss this kind of combination, we aim to further clarify this subject to provide the best practice for our patients.

## Background

The introduction of the laparoscopic surgery opened the doors for a better medical management and improved the patient's quality of life and it became the standard procedure for many surgical indications. Laparoscopic cholecystectomy considered one of the first laparoscopic procedures to be considered safe and reliable for gallbladder diseases 1. With time, Ralph Ger introduced the first laparoscopic hernia repair in 1982 2. The revolution in laparoscopic hernia repair was upon the introduction of the transabdominal preperitoneal (TAPP) hernia repair in 1992 3 and the totally extraperitoneal hernia repair. Although statistically there are no differences between open and laparoscopic TAPP hernia repair according to a new meta‐analysis, the laparoscopic approach, especially TAPP, is better regarding postoperative pain and later complications 4. Normally, patients diagnosed with cholelithiasis and found to have inguinal hernia are scheduled to undergo two elective laparoscopic procedures in two different times, but those patients can be ideal to undergo one laparoscopic operation to treat the two separated surgical indications avoiding the consequences of each operation alone. The safety and consequences to perform laparoscopic cholecystectomy and hernioplasty simultaneously are still questionable 5. A few cases were mentioned in the literature using the combination of these two operations. We performed laparoscopic hernia repair (TAPP) and cholecystectomy simultaneously to assess the safety, intra‐ and postoperative complications as well as surgical site infection (SSI).

## Cases Presentations

The presented cases involve 30‐ and 35‐year‐old male, Caucasian patients suffering from right upper abdominal pain with vomiting but without fever or jaundice. The symptoms started the previous night. There is no history of medical diseases and no regular medication is taken. The patients were examined in Damascus Hospital during 2015. The physical examination indicated a right subcostal pain with negative Murphy's sign and a body temperature of 36.5 and 37.2, respectively. Laboratory tests showed leukocyte, CRP, and ESR within normal range. According to the ultrasound study (US), they were diagnosed with symptomatic cholelithiasis. The patients were known candidates to an elective inguinal hernia repair. In physical examination, both patients had a reducible bulging in the right inguinal area that is clearly visible with coughing without any signs of strangulation or incarceration. The hernia was diagnosed later as a right direct inguinal hernia. The examination of the collateral side was normal. An informed consent was obtained from patients to perform cholecystectomy and repair the hernia laparoscopically at the same time.

**Figure 1:**
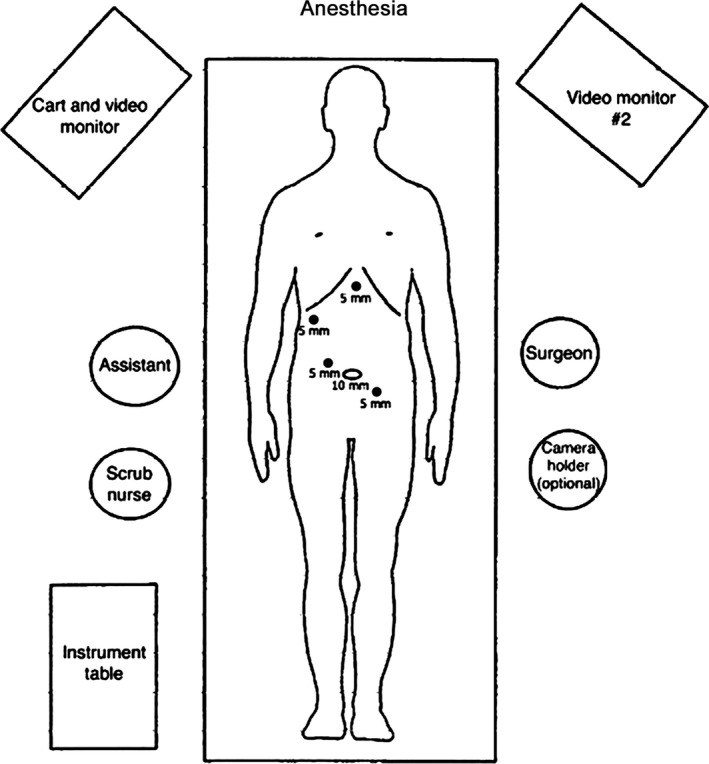
Showing the position of the surgeon, the monitoring screen, and positions of the trocars.

The two operations were performed in Damascus Hospital. Patients received a single dose of antibiotics (cefotaxime 1 g) before the operation and within 24 h after surgery to prevent postoperative complications 6. Under general anesthesia, the patients placed in Trendelenburg position and the operations began by insufflating carbon dioxide with a Veress needle through a subumbilical incision. The 10‐mm trocar (the optical trocar) was inserted at the umbilical level followed by three 5‐mm trocars: one right subcostal at the level of the middle clavicular line, one 2 cm right and above the umbilicus, and one epigastric (Fig. 1). After clear vision of the gallbladder, a standard cholecystectomy was performed first without perforating the gallbladder, and a drain was inserted under the liver that is removed after 24 h when no signs of bleeding or biliary leakage was obtained.

**Figure 2:**
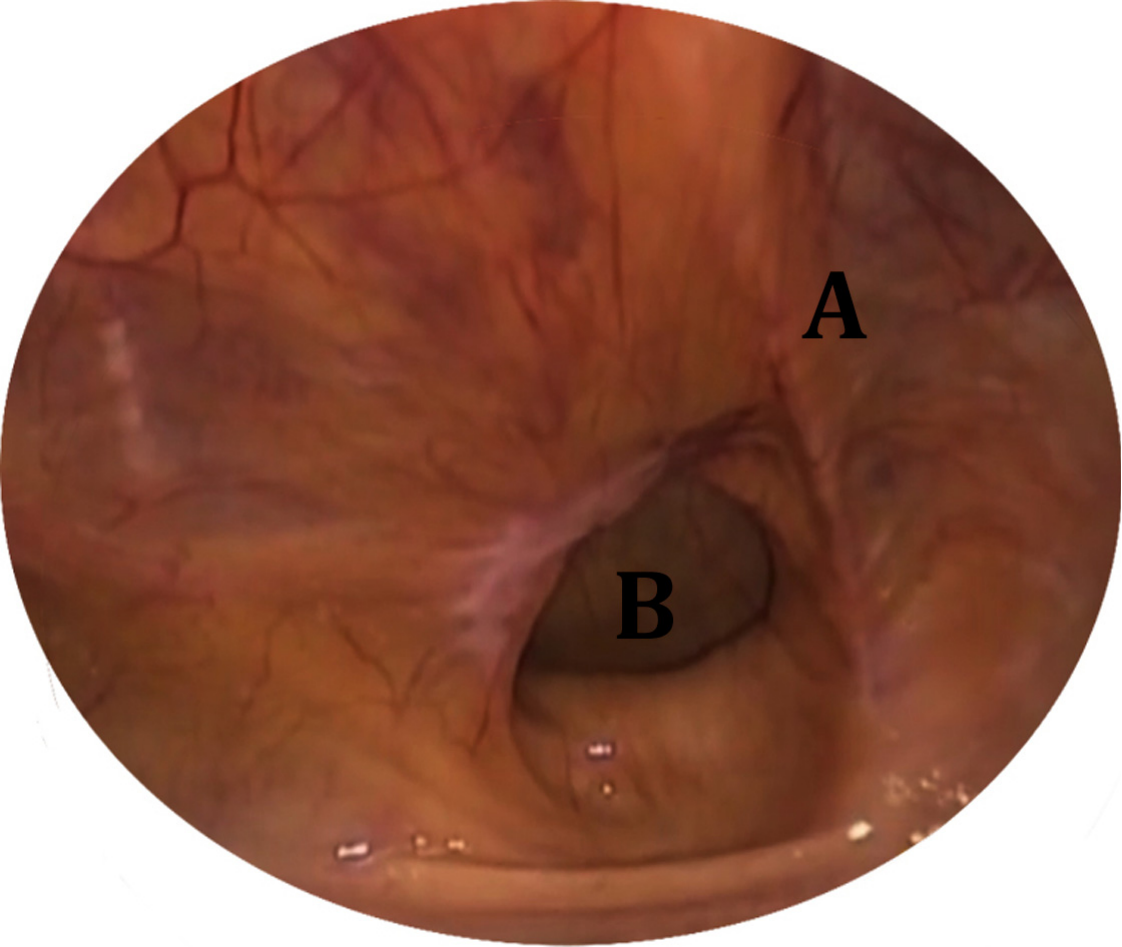
(A) right medial umbilical ligament, (B) right direct inguinal hernia.

**Figure 3:**
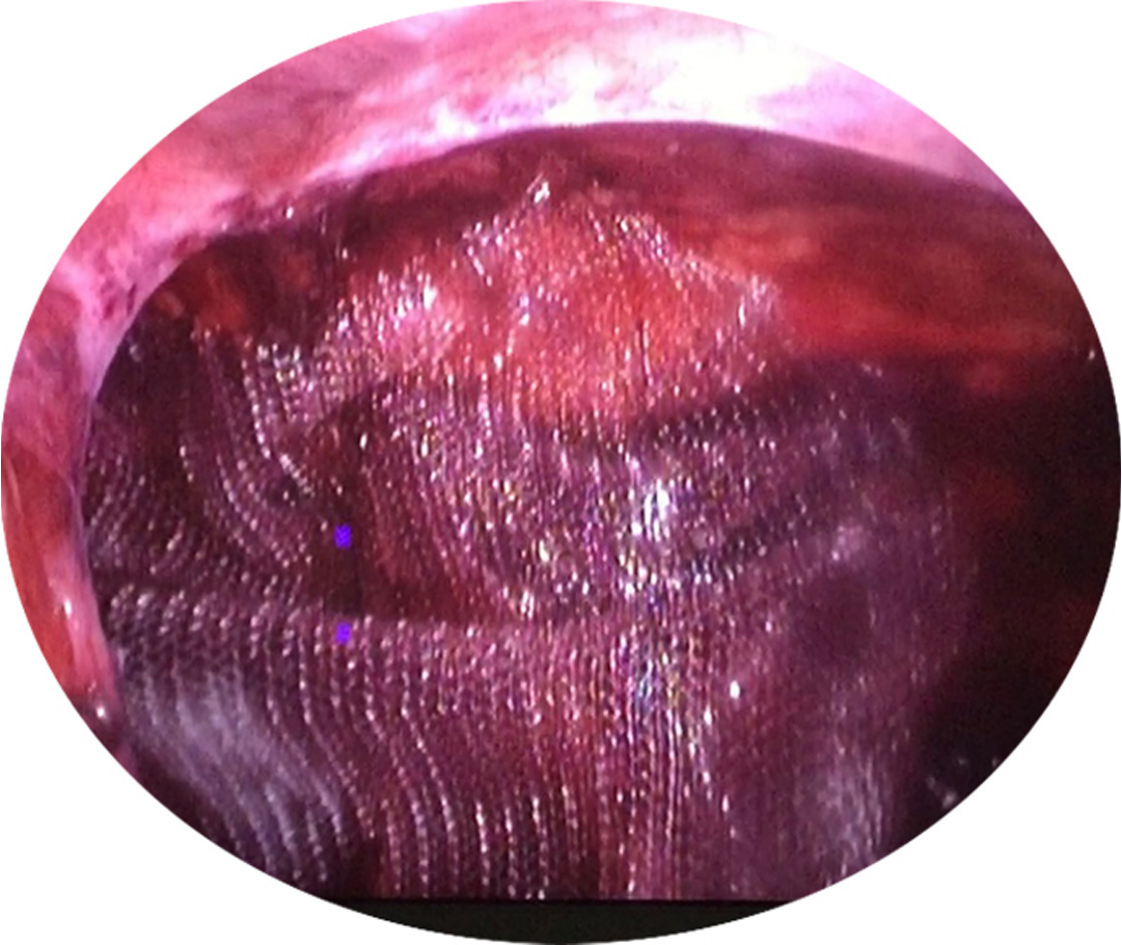
A 10 × 15 cm polypropylene mesh.

**Figure 4:**
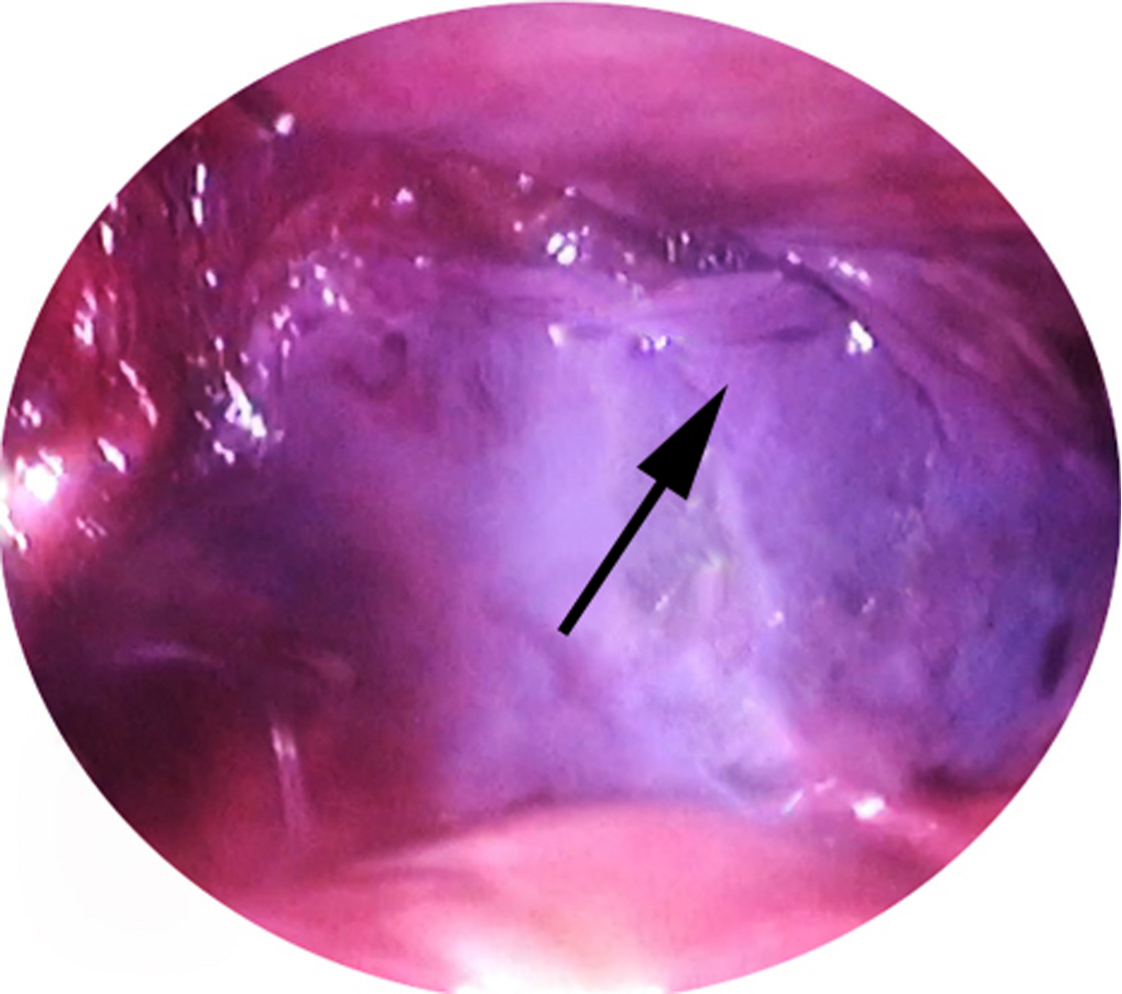
The preperitoneal space closed with continuous sutures.

The patients placed in reverse Trendelenburg position to perform the hernia repair. An extra trocar was inserted 2 cm left and below the umbilicus to offer a better control (Fig. 1). Under optical vision, a right direct inguinal hernia was diagnosed in both patients (Fig. 2). The anatomical structures were identified and the hernia sac was removed. A 10 × 15 cm polypropylene mesh was secured using staplers (Fig. 3). The preperitoneal space was closed using continuous sutures (Fig. 4). The procedure followed the Guidelines of the International Endohernia Society (IEHS) 7.

The patients were transferred to the surgical ward for monitoring and they stayed for 1 day. The two operations were done with no intra‐ or postoperative complication during the hospital stay. Both patients were hospitalized for 1 day. Follow‐up was made by senior residents at the hospital stay and later at the outpatient's clinic at 30 and 90 days and none of the patients experienced symptoms of infection nor SSI was noted during the examination.

## Discussion

Only two cases mentioned in PubMed describing the results of simultaneous laparoscopic cholecystectomy with laparoscopic inguinal hernia repair, using TAPP technique 5, 8. Although they confirmed the safety of this combination, only Sarli et al. illustrated the comparison versus open technique for hernia repair regarding the intra‐ and postoperative complications, pain after surgery, and followed the patients more than 48 h to observe the possible complications such as SSI which is one of the major concerns in these kinds of operations 8. In our cases, we amplify the safety of performing these two surgical indications in one operation in the areas of surgical technique, intra‐ and postoperative complications, in addition to the late complications; especially SSI. The assessment of recurrence requires more follow‐ups that were not applicable in our patients. The benefit of this combination is not only because it is safe but also protects the patient from being exposed twice to a general anesthesia and to prevent the possible complications of open hernia repair like pneumoperitoneum. On the other hand, it is not recommended to perform such a combination in an acute setting such as acute cholecystitis or incarcerated hernia due to high risk of infection that may affect the mesh and the complexity of this operation. Unlike the cases described by Lehmann et al. that repaired the inguinal hernia first to protect the mesh from contacting the bile 5. In our two cases, the gallbladder was removed before the hernia was repaired because the majority of cholecystectomies, due to a gallstone disease, are clean operations and would not affect the sterilization of the mesh. A study released by Abeysuriya et al. stated that the isolated bacteria from the bile showed that only the pigmented stone‐containing bile is associated with bacteria 9. These kinds of bacteria are usually normal flora, according to a new systematic review and meta‐analysis; prophylactic antibiotic can reduce effectively postoperative infections including SSI 6. Further studies must be conducted to reach a solid results and guidelines regarding this combination.

## Conclusion

The combination of the two procedures is safe in certain selective cases and does not increase the possibility of postsurgical infections especially when prophylactic antibiotics are administrated to reduce the chance of SSI. With experienced surgeons, the operations can be done uneventfully but we need more studies to confirm our results and to reach guidelines applicable to all surgeons.

## Authorship

SA: The first author, wrote the manuscript, contributed to the analysis of the data and literature review; MBA: Contributed to the concept, analysis, and literature review and was the surgeon in the patient's operations.

## Conflicts of Interest

The authors declared that they have no competing interests.
